# Pediatric Respiratory Syncytial Virus Hospitalizations, 2017-2023

**DOI:** 10.1001/jamanetworkopen.2024.16077

**Published:** 2024-06-11

**Authors:** Tiffany Fitzpatrick, Sarah A. Buchan, Sanjay Mahant, Longdi Fu, Jeffrey C. Kwong, Therese A. Stukel, Astrid Guttmann

**Affiliations:** 1 Public Health Ontario, Toronto, Ontario, Canada; 2 Dalla Lana School of Public Health, University of Toronto, Toronto, Ontario, Canada; 3 Centre for Vaccine Preventable Diseases, University of Toronto, Toronto, Ontario, Canada; 4 ICES, Toronto, Ontario, Canada; 5 Institute for Health Policy, Management, and Evaluation, University of Toronto, Toronto, Ontario, Canada; 6 The Hospital for Sick Children, Toronto, Ontario, Canada; 7 Department of Family and Community Medicine, University of Toronto, Toronto, Ontario, Canada; 8 University Health Network, Toronto, Ontario, Canada; 9 Edwin S. H. Leong Centre for Healthy Children, University of Toronto, Toronto, Ontario, Canada

## Abstract

**Importance:**

Respiratory syncytial virus (RSV) transmission was disrupted worldwide following the COVID-19 pandemic, and further study is required to better understand these changes.

**Objective:**

To compare observed and expected RSV hospital and intensive care unit (ICU) admission rates and characteristics of admitted children during the 2021-2022 and 2022-2023 seasons.

**Design, Setting, and Participants:**

A population-based cohort study of all children aged younger than 5 years in Ontario, Canada, July 1, 2017, through March 31, 2023, was conducted.

**Exposures:**

Individual and neighborhood-level sociodemographic and clinical characteristics were identified from administrative data, including age, palivizumab eligibility, complex medical conditions, rurality, and living in a marginalized neighborhood.

**Main Outcomes and Measures:**

The main outcome was RSV-associated hospitalization. Secondary outcomes included ICU admissions, mechanical ventilation, extracorporeal membrane oxygenation, and in-hospital death. Poisson generalized estimating equations were used to model weekly age- and sex-specific hospitalization rates and estimate expected rates in the postpandemic era; adjusted rate ratios (RRs) and 95% CIs are reported.

**Results:**

This cohort study included approximately 700 000 children per study year. Compared with prepandemic years (2017-2018, 2018-2019, and 2019-2020), the 2021-2022 RSV season peaked slightly earlier, but overall admission rates were comparable (289.1 vs 281.4-334.6 per 100 000, or approximately 2000 admissions). The 2022-2023 season peaked a month earlier and resulted in more than twice as many hospitalizations (770.0 per 100 000; n = 4977 admissions). The proportion of children admitted to an ICU in 2022-2023 (13.9%) was slightly higher than prepandemic (9.6%-11.4%); however, the population-based rate was triple the prepandemic levels (106.9 vs 27.6-36.6 per 100 000 children in Ontario). With the exception of palivizumab-eligible children, all sociodemographic and health status characteristics were associated with lower-than-expected RSV hospitalization rates in 2021-2022. In contrast, older age of patients was associated with higher-than-expected rates in 2022-2023 (ie, 24-59 months: RR, 1.90; 95% CI, 1.35-2.66).

**Conclusions and Relevance:**

There were notable differences in RSV epidemiologic characteristics in Ontario following the COVID-19 pandemic. It is not yet clear whether and how long atypical RSV epidemics may persist. Clinicians and program planners should consider the potential for ongoing impacts to health care capacity and RSV immunization programs.

## Key Points

**Question:**

Were there changes in the epidemiologic characteristics of pediatric respiratory syncytial virus (RSV) in Ontario, Canada, following the COVID-19 pandemic?

**Findings:**

In this cohort study of 700 000 children younger than 5 years per study year, the 2021-2022 RSV season peaked slightly earlier than expected, but overall admission rates were comparable with prepandemic seasons; in contrast, the 2022-2023 season peaked earlier and resulted in more than twice as many hospitalizations. There were notable differences in sociodemographic and clinical characteristics of children admitted after vs before the pandemic.

**Meaning:**

These findings suggest the need for ongoing surveillance to assess the persistence of atypical RSV epidemics and studies examining any implications this may have for capacity planning and immunization programs.

## Teaser

This cohort study examines the hospitalization rates associated with respiratory syncytial virus infection during the 2021-2022 and 2022-2023 seasons compared with the pre-COVID-19 2017-2020 seasons in Ontario, Canada.

## Introduction

Respiratory syncytial virus (RSV) is the leading cause of lower respiratory illness and hospitalization globally among children younger than 5 years.^1^ While nearly every child acquires at least 1 RSV infection before their second birthday, infants younger than 6 months and children with specific cardiopulmonary conditions are at greatest risk of severe disease.^2^ Historically, RSV followed a predictable annual seasonal pattern; for example, in the Northern hemisphere and temperate Southern regions, RSV activity consistently increased each year during the winter months (ie, peaking in December to January in the Northern hemisphere and June to July in Southern regions).^3,4^ However, nonpharmaceutical interventions implemented in response to the COVID-19 pandemic, such as social distancing, mask mandates, travel restrictions, and school closures, also interrupted the transmission of other respiratory viruses, including RSV.^3,5,6^ In many regions, this led to a rapid reduction in RSV cases in March 2020, followed by the near absence of RSV during the 2020-2021 season.^3,5,6,7^

Following the relaxation of pandemic measures in late 2020, many areas throughout the Southern hemisphere observed the resurgence of seasonal respiratory viruses; Australia was among the first to report an out-of-season return of RSV in late 2020.^3^ Many countries experienced earlier-than-expected 2021-2022 RSV seasons, followed by a persistence of RSV activity throughout the summer of 2021.^5^ During the 2022-2023 season, RSV gained widespread public attention as a historically high surge in RSV-related disease, combined with influenza and COVID-19, led to a “tripledemic,” overwhelming many pediatric health care systems worldwide.^7,8^ In some communities, pediatric health systems were so strained as to require emergency measures. For example, in the US, some states declared states of emergency or called on the National Guard,^9,10^ and several countries in South America temporarily reinstated mask mandates and school closures.^11^ In response to the earlier-than-expected start of the 2021-2022 and 2022-2023 seasons, many jurisdictions, including Ontario, also initiated their RSV immunoprophylaxis programs weeks earlier than typical.^12,13^ In addition to being much earlier and more intense than expected, there were also questions as to whether more children were presenting with severe forms of RSV-related disease in 2022-2023 than usual.^9,10^

A detailed understanding of whether there were changes in the clinical and sociodemographic characteristics of children admitted to hospitals and intensive care units (ICUs) post pandemic, particularly during the 2022-2023 season, is currently lacking. Unlike COVID-19, RSV is not a notifiable disease in most jurisdictions.^14^ Thus, the real-time, population-based surveillance data needed to support rapid investigations of changes in RSV seasonality and epidemiologic factors are limited. Some national sentinel surveillance and population-based administrative studies have reported high-level observations from the 2021-2022 season^4,7,15,16^; however, those examining the 2022-2023 season are so far limited to single-center studies and a handful of multicenter hospital-based studies, and largely only capture the first few months of the season.^4,8,16,17,18^ We used a population-based repository of linked health and sociodemographic administrative data to compare the observed and expected rates of RSV-related hospitalizations and ICU admissions among children younger than 5 years in Ontario, Canada, in the 2 seasons following the COVID-19 pandemic (ie, 2021-2023), compared with a baseline (2017-2020), by clinical and sociodemographic characteristics.

## Methods

### Study Population and Data Sources

All children younger than 5 years living in Ontario during the study period (July 1, 2017, through March 31, 2023, inclusive) were identified from a population registry of individuals eligible for provincial (universal) health insurance. Respiratory syncytial virus seasons were defined as July 1 through June 30 of the following year to capture typical annual RSV seasonal patterns. The use of data in this project was authorized under section 45 of Ontario’s Personal Health Information Protection Act and exempt from research ethics board review. This study followed the Strengthening the Reporting of Observational Studies in Epidemiology (STROBE) reporting guideline.

All RSV-related hospitalizations occurring across Ontario were identified using a highly sensitive and specific definition based on health administrative data.^19^ Sociodemographic and health status characteristics of children were determined at index (ie, RSV admission date or July 1 of a given year) using health- and census-based databases. Datasets were linked using unique encoded identifiers and analyzed at ICES (eMethods in Supplement 1).^20^

### Statistical Analysis

Annual population-based hospitalization, ICU, and mechanical ventilation (including invasive and noninvasive) rates were determined based on the observed number of children admitted or requiring mechanical ventilation and population-based denominators including all children younger than 5 years as determined on July 1 of each year.

Weekly age- and sex-specific RSV admission rates were calculated using age- and sex-specific population denominators, as determined on July 1 of each year. We used Poisson generalized estimating equations to model prepandemic weekly hospitalization rates and estimate expected weekly rates in the pandemic era, defined as March 1, 2020, onward. In addition to age and sex, the model included prepandemic secular trend and monthly indicators. The number of RSV admissions was set to 0.001 for weeks with 0 RSV admissions to mitigate convergence issues.

Two-sided 95% CIs were estimated by applying the linear combination of prepandemic regression coefficients to pandemic era data and exponentiating.^21^ The relative change in observed vs expected admissions following the pandemic onset was estimated as adjusted rate ratios (RRs) of observed vs expected by exponentiating the difference between observed and expected log rates and CIs in the pandemic era, as further described in previous work examining the impact of SARS-CoV-1– and SARS-CoV-2–associated restrictions on health care use in Canada.^21,22,23^ Model performance in the prepandemic period was assessed via normalized root mean squared error, where 0 suggests a perfect fit to the observed data and values less than 0.5 indicate a relatively good fit (eTable 1 in Supplement 1). All analyses were performed using SAS, version 9.4 (SAS Institute Inc).

## Results

Approximately 700 000 children younger than 5 years were included in each year of the study, with 2008 to 2421 children admitted annually for RSV prepandemic, 1972 in 2021-2022 and 4977 in 2022-2023 (Table). Only 11 RSV hospitalizations and 7 ICU admissions occurred during the 2020-2021 season. There were clear changes in the age distribution of children admitted for RSV during the postpandemic seasons, particularly during the 2022-2023 season. Before the pandemic, the mean (SD) age of children hospitalized with RSV was 10.8 (12.64) to 12.6 (13.32) months, and the median (IQR) age was 5 (2-16) to 7 (2-19) months; in 2022-2023, the mean (SD) age was 16.1 (15.46) months and the median (IQR) age was 11 (3-26) months (Table).

**Table.** Characteristics of Children Younger Than 5 Years Hospitalized for RSV-Related Illness in Ontario, 2017-2018 Through 2022-2023^a^

Characteristic	RSV season^b^				
	2017-2018	2018-2019	2019-2020	2021-2022	2022-2023
Population size	713 547	712 233	708 633	682 201	646 401
No. hospitalized	2008	2383	2421	1972	4977
Overall admission rate, per 100 000 children	281.4	334.6	341.6	289.1	770.0
Age at time of admission, No. (%), mo					
<1	547 (27.2)	657 (27.6)	561 (23.2)	585 (29.7)	958 (19.2)
2-3	303 (15.1)	393 (16.5)	372 (15.4)	288 (14.6)	635 (12.8)
4-5	199 (9.9)	198 (8.3)	211 (8.7)	141 (7.2)	367 (7.4)
6-11	315 (15.7)	321 (13.5)	319 (13.2)	195 (9.9)	609 (12.2)
12-23	368 (18.3)	446 (18.7)	534 (22.1)	337 (17.1)	1028 (20.7)
24-35	148 (7.4)	203 (8.5)	219 (9.0)	231 (11.7)	620 (12.5)
36-59	128 (6.4)	165 (6.9)	205 (8.5)	195 (9.9)	760 (15.3)
Mean (SD)	10.81 (12.64)	11.26 (12.88)	12.56 (13.32)	12.85 (14.29)	16.08 (15.46)
Median (IQR)	5 (2-16)	5 (2-17)	7 (2-19)	5 (2-22)	11 (3-26)
Male sex, No. (%)	1118 (55.7)	1287 (54.0)	1338 (55.3)	1096 (55.6)	2755 (55.4)
Rural residence, No. (%)	209 (10.4)	283 (11.9)	250 (10.3)	262 (13.3)	594 (11.9)
Neighborhood income quintile, No. (%)					
1 (Lowest income)	455 (22.7)	551 (23.1)	540 (22.3)	392 (19.9)	1068 (21.5)
2	422 (21.0)	462 (19.4)	429 (17.7)	346 (17.5)	909 (18.3)
3	395 (19.7)	484 (20.3)	510 (21.1)	380 (19.3)	1022 (20.5)
4	369 (18.4)	500 (21.0)	478 (19.7)	451 (22.9)	1001 (20.1)
5 (Highest income)	352 (17.5)	359 (15.1)	427 (17.6)	352 (17.8)	824 (16.6)
Unknown	15 (0.7)	27 (1.1)	37 (1.5)	51 (2.6)	153 (3.1)
Material resources quintile, No. (%)					
1 (Least marginalized)	361 (18.0)	447 (18.8)	478 (19.7)	379 (19.2)	924 (18.6)
2	367 (18.3)	456 (19.1)	479 (19.8)	417 (21.1)	945 (19.0)
3	370 (18.4)	425 (17.8)	434 (17.9)	345 (17.5)	943 (18.9)
4	373 (18.6)	444 (18.6)	390 (16.1)	343 (17.4)	895 (18.0)
5 (Most marginalized)	498 (24.8)	553 (23.2)	568 (23.5)	421 (21.3)	1031 (20.7)
Unknown	39 (1.9)	58 (2.4)	72 (3.0)	67 (3.4)	239 (4.8)
Any comorbidities at birth, No. (%)					
Major congenital anomaly	222 (11.1)	247 (10.4)	236 (9.7)	212 (10.8)	439 (8.8)
Congenital heart disease	7 (0.3)	19 (0.8)	18 (0.7)	12 (0.6)	25 (0.5)
Chronic lung disease	92 (4.6)	102 (4.3)	99 (4.1)	99 (5.0)	159 (3.2)
Trisomy 21	19 (0.9)	14 (0.6)	26 (1.1)	13 (0.7)	35 (0.7)
Any chronic medical condition, No. (%)	340 (16.9)	406 (17.0)	431 (17.8)	346 (17.5)	796 (16.0)
Born preterm (<37 wk), No. (%)	293 (14.6)	404 (17.0)	374 (15.4)	312 (15.8)	746 (15.0)
Palivizumab eligibility, No. (%)					
Clearly eligible	66 (3.3)	88 (3.7)	56 (2.3)	57 (2.9)	106 (2.1)
Possibly eligible	75 (3.7)	66 (2.8)	57 (2.4)	65 (3.3)	111 (2.2)
Ineligible	1867 (93.0)	2229 (93.5)	2308 (95.3)	1850 (93.8)	4760 (95.6)
Hospitalization outcomes					
Length of stay, mean (SD), d	3.75 (5.12)	4.08 (9.64)	3.78 (6.00)	3.96 (9.25)	4.16 (5.47)
ICU admission, No. (%)	197 (9.8)	261 (11.0)	233 (9.6)	224 (11.4)	691 (13.9)
ICU length of stay, mean (SD), d	1.14 (0.51)	1.17 (0.46)	1.23 (0.58)	1.26 (0.85)	1.15 (0.50)
Overall ICU admission rate, per 100 000 children (population-based)	27.61	36.65	32.88	32.83	106.90
Mechanical ventilation use, No. (%)	85 (4.2)	104 (4.4)	101 (4.2)	65 (3.3)	235 (4.7)
Time requiring mechanical ventilation, mean (SD), d	1.29 (0.61)	1.27 (0.84)	1.37 (0.80)	1.25 (0.50)	1.23 (0.53)
Overall mechanical ventilation rate, per 100 000 children (population-based)	11.91	14.60	14.25	9.53	36.36
Required ECMO, No. (%)^c^	0	<6	0	0	<6
In-hospital death, No. (%)^c^	0	<6	<6	<6	<6

Abbreviations: ECMO, extracorporeal membrane oxygenation; ICU, intensive care unit; RSV, respiratory syncytial virus.

^a^ Eleven RSV hospitalizations and 7 ICU admissions occurred during the 2020-2021 season; data not shown given small cell sizes.

^b^ RSV season defined as July 1 through June 30 of the subsequent year.

^c^ Cell sizes less than 6 have been suppressed.

**Figure 1.**
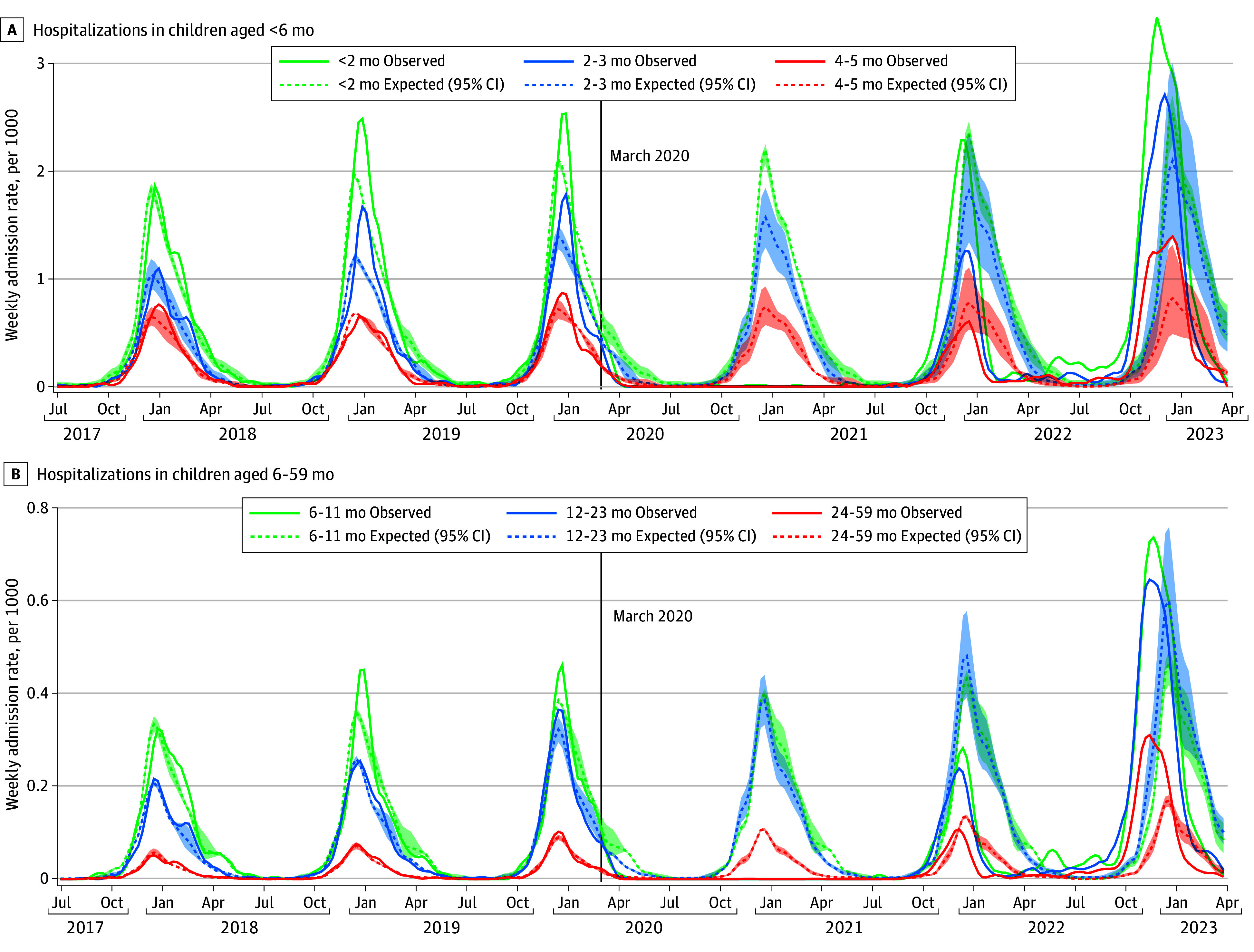
Observed and Expected Weekly Age-Specific Respiratory Syncytial Virus (RSV)–Associated Hospitalization Rates (per 1000 Children) Rates from July 1, 2017, through March 31, 2023, in children younger than 6 months (A) and 6 to 59 months (B). Shaded area indicates 95% CI.

**Figure 2.**
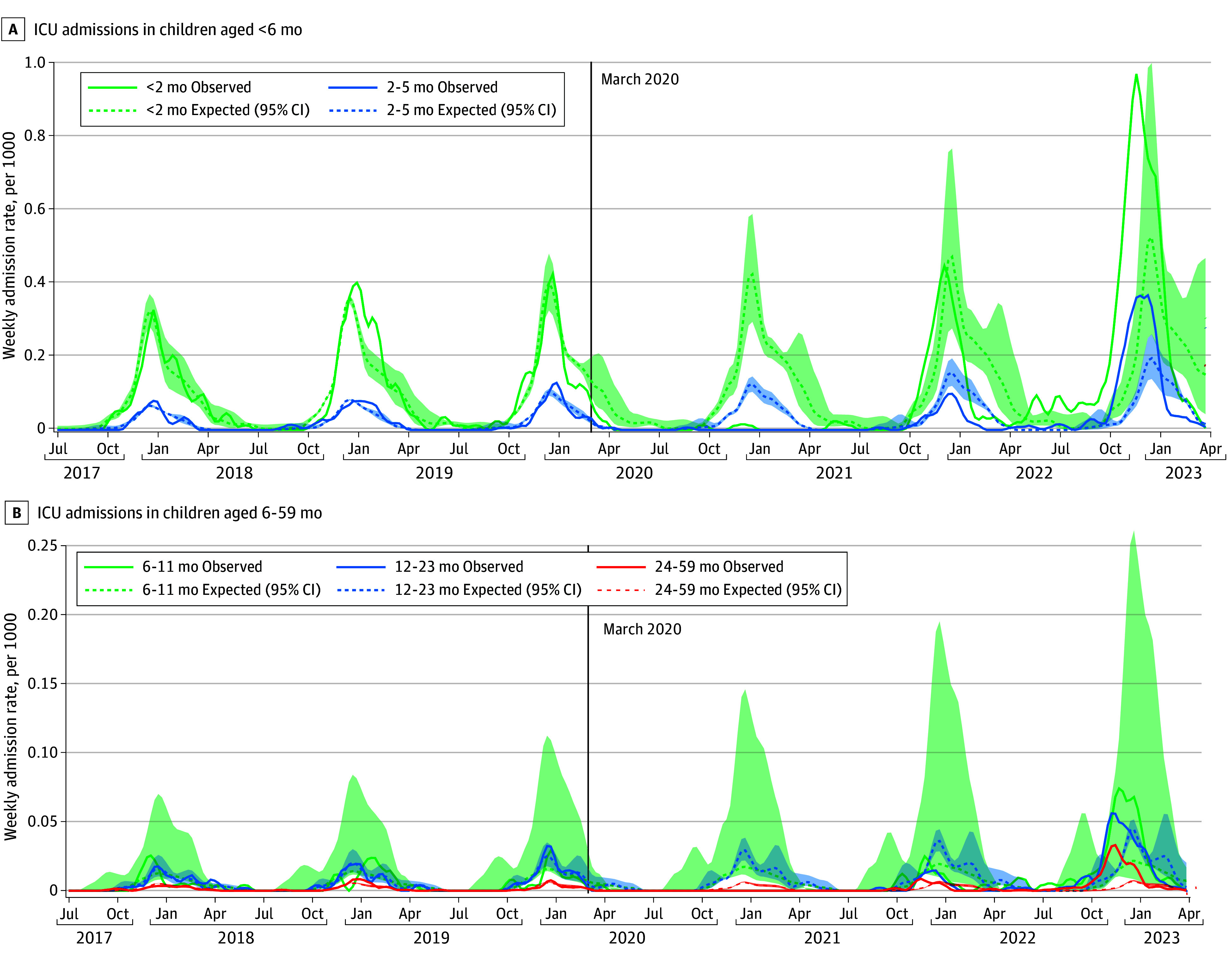
Observed and Expected Weekly Age-Specific Respiratory Syncytial Virus–Associated Intensive Care Unit Admission Rates (per 1000 Children) Rates from July 1, 2017, through March 31, 2023, in children younger than 6 months (A) and 6 to 59 months (B). Shaded area indicates 95% CI.

Compared with prepandemic years (2017-2018, 2018-2019, and 2019-2020), the 2021-2022 RSV season peaked slightly earlier (early December compared with mid-December), but overall hospitalization rates were comparable (289.1 vs 281.4-334.6 per 100 000 over the entire season; approximately 2000 admissions) (Table and Figure 1); with the exception of children aged younger than 2 months, the peak number of observed weekly admissions was notably lower than expected for all age groups. In contrast, the 2022-2023 season peaked over a month earlier and resulted in more than twice as many cumulative hospitalizations (770.0 per 100 000, n = 4977 admissions). Moreover, the peak number of weekly admissions in 2022-2023 was distinctly higher than expected for all age groups. Additionally, there was an out-of-season continuation of RSV admissions over the spring and summer of 2022 among all age groups. Similar changes in seasonal timing and magnitude were noted for ICU admissions (Figure 2).

A higher proportion of children was admitted to ICU during 2021-2022 (11.4%) and, particularly, 2022-2023 (13.9%), compared with the prepandemic seasons (9.6%-11.0%) (Table); this is also reflected in the 3-fold increase in ICU admission rates (27.6-36.6 per 100 000 prepandemic vs 106.9 in 2022-2023). Similarly, a slightly higher proportion of children admitted in 2022-2023 required mechanical ventilation (4.7% compared with 4.2%-4.4% in prepandemic years), whereas slightly fewer required mechanical ventilation in 2021-2022 (3.3%). The rate of mechanical ventilation use was also 2- to 3-fold higher in 2022-2023 compared with prepandemic years (11.9-14.6 per 100 000 prepandemic vs 36.4 in 2022-2023). There were no significant differences in the length of hospital or ICU stay, number of days for which mechanical ventilation was required, or more severe outcomes (ie, extracorporeal membrane oxygenation use and in-hospital deaths).

**Figure 3.**
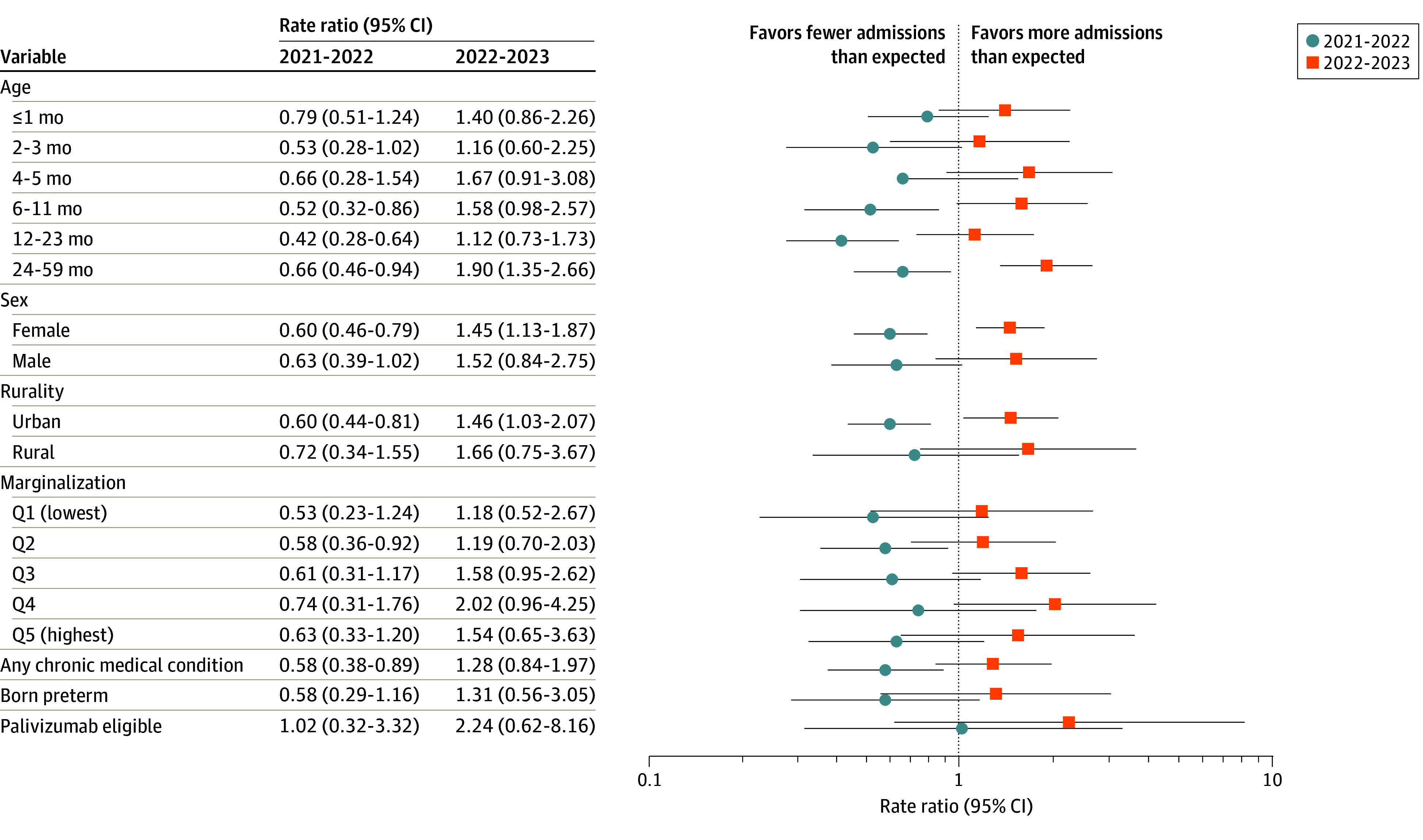
Adjusted Rate Ratio of Observed vs Expected Respiratory Syncytial Virus–Associated Hospitalizations According to Sociodemographic and Medical Characteristics Hospitalizations during the 2021-2022 and 2022-2023 vs the 2017-2019 and 2019-2020 seasons. Q indicates quintile.

With the exception of palivizumab-eligible children (RR, 1.02; 95% CI, 0.32-3.32), all children experienced lower-than-expected hospitalization rates during the 2021-2022 season, compared with the prepandemic seasons (Figure 3; eTable 2 in Supplement 1). This was most apparent for children aged 12 to 23 months (RR, 0.42; 95% CI, 0.28-0.64); however, both clinically and statistically significant changes were noted for children with several other characteristics, including children aged 6 to 11 months, those diagnosed with any complex medical condition, those living in areas with lower (quintile 2) marginalization, and females. In contrast, all children were more likely to be hospitalized with RSV in 2022-2023, compared with the prepandemic seasons. The largest of these differences was observed among palivizumab-eligible children (RR, 2.24; 95% CI, 0.62-8.16), children living in more marginalized neighborhoods (eg, quintile 4: RR, 2.02; 95% CI, 0.96-4.25), and older children (ie, 24-59 months: RR, 1.90; 95% CI, 1.35-2.66), albeit not all were statistically significant. While clinically important differences were noted for many characteristics, the only statistically significant changes were observed among children aged 24 to 59 months, children residing in rural areas (RR, 1.46; 95% CI, 1.03-2.07), and girls (RR, 1.45; 95% CI, 1.13-1.87).

**Figure 4.**
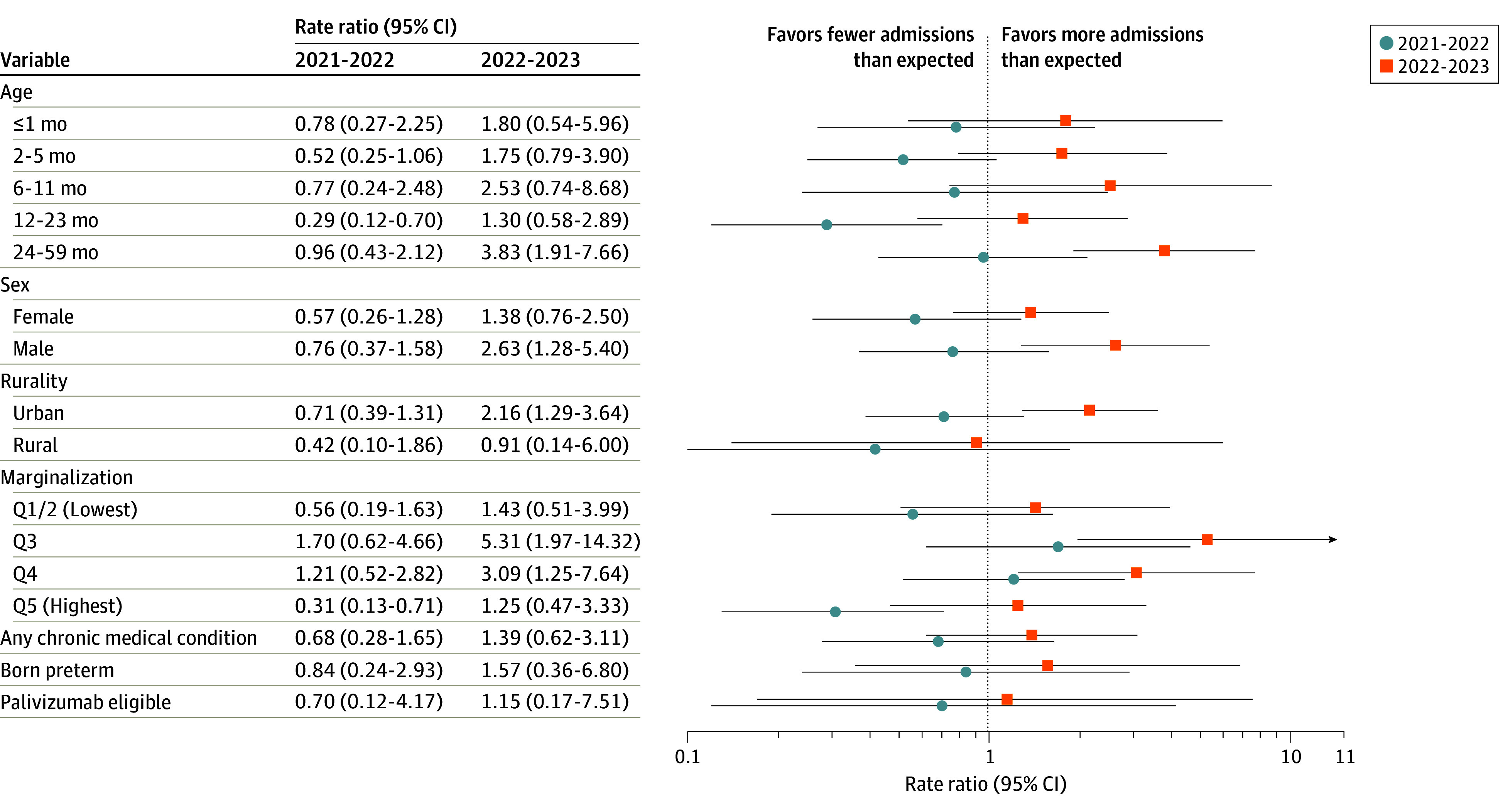
Adjusted Rate Ratio of Observed vs Expected Respiratory Syncytial Virus–Associated Intensive Care Unit Admissions According to Sociodemographic and Medical Characteristics Intensive care unit admissions during the 2021-2022 and 2022-2023 vs the 2017-2019 and 2019-2020 seasons. The x-axis is shown on log scale. Q indicates quintile.

With the exception of children living in moderately marginalized communities (quintiles 3 and 4), most children were less likely than expected to be admitted to ICU during the 2021-2022 season, compared with prepandemic seasons (Figure 4; eTable 2 in Supplement 1). In contrast, with the exception of those living in rural communities (RR, 0.91; 95% CI, 0.14-6.00), children of all examined characteristics were more likely than expected to be admitted to ICU in 2022-2023. Many of these changes were both clinically and statistically significant, for example, quintile 3 neighborhood marginalization (RR, 5.31; 95% CI, 1.97-14.32), age 24-59 months (RR, 3.83; 95% CI, 1.91-7.66), quintile 4 neighborhood marginalization (RR, 3.09; 95% CI, 1.25-7.64), male sex (RR, 2.63; 95% CI, 1.28-5.40), and urban residence (RR, 2.16; 95% CI, 1.29-3.64).

## Discussion

There were clear changes in the timing and intensity of annual RSV epidemics in Ontario following the COVID-19 pandemic: there was a near absence of RSV in 2020-2021; a slightly earlier and less burdensome season in 2021-2022, followed by an atypical out-of-season persistence of RSV admissions throughout 2022 and a much earlier-than-expected and historically intense season in 2022-2023. There was also some evidence to suggest that hospitalized children experienced a slightly higher severity of disease in 2022-2023 compared with prepandemic years, as indicated by the proportion and rate of ICU admissions and mechanical ventilation use. In all, there were abnormally high burdens placed on the pediatric health care system in Ontario during the 2022-2023 season due to the intensity of this RSV surge. In addition to these changes in seasonality, burden, and severity, the overall epidemiologic characteristics of each RSV season were markedly different post pandemic. For example, there was a substantial shift in the age of children admitted with RSV, particularly in 2022-2023; on average, children hospitalized in 2022-2023 were approximately 4 to 5 months older than expected. There were also significant differences in the characteristics of children admitted in the prepandemic vs postpandemic seasons on the basis of sex, rurality, neighborhood-level marginalization, and health status, such as diagnosis of any complex medical condition, palivizumab program eligibility, and prematurity.

Many other jurisdictions observed similar changes in the timing and intensity of RSV activity postpandemic, particularly following the relaxation of nonpharmaceutical interventions.^3,24^ For example, the US Centers for Disease Control and Prevention and other multicenter studies have reported that the 2021-2022 RSV season started—and peaked—several months earlier than expected and persisted over the spring and summer months.^4,8^ While few analyses of the 2022-2023 season have yet been published,^4,7,18,25,26,27^ a Danish national study, which included data up to January 2023, reported that older children were considerably more likely to be admitted during both the 2021-2022 and 2022-2023 seasons.^25^ The authors also estimated a significant increase in mechanical ventilation rates among children younger than 5 years admitted for RSV in 2021-2022 (2.3-fold) and 2022-2023 (1.5-fold). A large multicenter US study capturing bronchiolitis admissions in children younger than 2 years through June 2023 similarly observed that children admitted during the 2022-2023 season were older, more likely to be admitted to an ICU, and more likely to receive noninvasive ventilation.^17^ Moreover, a single-center study from British Columbia, Canada, which included all youth younger than 18 years with a laboratory test positive for RSV performed by the single pediatric hospital in British Columbia through May 2023 also reported a considerable increase in the age of children admitted for RSV in postpandemic years: a median of 17.8 in 2021-2022 and 19.6 months in 2022-2023, compared with 8.7 to 12.4 in 2017-2018 to 2019-2020.^26^ There was limited evidence to suggest an increase in severity of postpandemic RSV cases in British Columbia, as indicated by ICU admissions, mechanical ventilation use, and supplemental oxygen use.^26^ We also observed that older children were more likely to be admitted post pandemic and found some evidence to suggest an increase in severity in 2022-2023, similar to the Danish^25^ and US^17^ studies.

While not statistically significant, proportionally fewer children in British Columbia admitted during postpandemic seasons were born prematurely (<29 or <37 weeks’ gestation) or had underlying respiratory or cardiac conditions.^26^ We similarly observed a decrease in admissions for children with these characteristics during the 2021-2022 season; however, Ontarian children with these characteristics experienced relatively higher-than-usual admission rates in 2022-2023. In British Columbia, no changes were observed related to sex or whether children resided in the metropolitan Vancouver area^26^; in contrast, we observed differences in relative rates among urban and rural children, particularly with respect to ICU admissions. Most single-center and multicenter hospital-based studies often underrepresent children living outside of urban centers, where tertiary pediatric hospitals are most commonly located; as our study is uniquely population-based, our findings are representative of both urban- and rural-residing children. Although, to our knowledge, no other studies have investigated changes related to the wider sociodemographic characteristics included in this study, the changes we observed related to rurality and marginalization likely reflect the increased likelihood of transmission in more densely populated areas and associations with other sociodemographic factors known to increase the risk of transmission of RSV, SARS-CoV-2, and other respiratory viruses (eg, larger household size, daycare attendance, and having essential workers in the household).^28,29^

Work has only begun to understand the specific mechanisms through which the COVID-19 pandemic impacted the seasonality and epidemiologic characteristics of RSV, although several plausible theories have been proposed.^5^ Most prominent is that the effective disruption of RSV transmission through pandemic mitigation measures predictably resulted in a cohort of newborns without direct exposure to the virus and, thus, a larger proportion of children were susceptible to RSV the following year, once viral activity increased.^30^ Moreover, considerable antibody waning for RSV occurs after 6 to 12 months; thus, achieving optimal passive antibody levels in infants requires repeated maternal RSV exposure.^31^ Serologic data from British Columbia suggest a population-level deficiency in RSV immunity occurred in 2020 among both women of childbearing age and infants^31^; a Dutch study of individuals aged 1 to 89 years found similar reductions in 2020 and 2021.^32^ These immunity changes have also been referred to as an immunity debt.^33,34,35^ Combined, these findings explain the consistent observation that older children were more likely to be hospitalized with RSV in recent years as a cohort of children aged 12 to 36 months would have been largely immunologically naive to RSV; while the first RSV infection is associated with increased disease severity, older children are more immunologically and physiologically mature and, thus, can better respond to an RSV infection than younger infants.^5^

### Limitations

Although our study provides a unique population-based perspective including detailed information on the sociodemographic and clinical characteristics of children admitted for RSV before vs after the COVID-19 pandemic and includes data spanning the 2022-2023 season, there are some limitations. As with other studies, this work is limited to children whose parents sought medical care for RSV. While Ontario has a universal health care system, it is possible that some parents did not seek care for their child; particularly, widespread news coverage of overwhelmed emergency departments may have deterred parents of less severely ill children from seeking medical care. Conversely, as thresholds to admit to hospitals may increase when bed supply is limited, it is more likely that some children who would have previously been admitted were not in 2022-2023, thereby resulting in an underestimate of the true magnitude of these differences in the 2022-2023 season; however, it is also likely that clinicians may have been more inclined to admit given concerns regarding a potential increase in disease severity. A considerable challenge for all studies is the incomparability of RSV surveillance data before vs after the pandemic resulting from testing practice changes.^4^ Prior to the pandemic, confirmation of RSV infection was less frequently sought as RSV treatment is mostly supportive. The COVID-19 pandemic has resulted in the broader availability and use of multiplex testing.^4^ Consequently, it is likely that more bronchiolitis and pneumonia admissions that would have been unspecified in earlier years are currently accurately classified as RSV related. However, we used an administrative data algorithm that does not explicitly require laboratory confirmation. Moreover, a multicenter US study that looked at changes in RSV-confirmed admissions, RSV-associated bronchiolitis, and unspecified bronchiolitis noted a considerable increase in RSV-associated admissions and bronchiolitis in 2022-2023, but no change in unspecified bronchiolitis, and a decrease in all RSV-associated and bronchiolitis admissions in 2021-2022.^4^ This suggests real decreases in RSV-related disease occurred in 2021-2022 and increases occurred in 2022-2023 that were not just artifacts of testing changes.

## Conclusions

In this population-based cohort study, we observed notable differences in the seasonality, burden, severity, and epidemiologic characteristics of RSV in each of the 3 seasons following the COVID-19 pandemic. Clinicians and program planners should consider that atypical RSV epidemics may continue into the future and plan for ongoing annual tripledemics of COVID-19, RSV, and influenza, which may continue to pose considerable strains on health care systems. Additionally, the potential for ongoing changes in the seasonality and epidemiologic characteristics of RSV in both the short- and long-term future should be considered in the delivery of seasonal prophylaxis and emerging vaccine products for infants, pregnant people, and older adults. Finally, the unexpected and widespread influences on seasonal respiratory viruses that followed the COVID-19 pandemic underscore the need for ongoing research to understand the impact of pandemic mitigation measures and the unique factors of transmission for common pathogens to ensure societies are better prepared to respond to future pandemics.

## Notes

Supplement 1. **eMethods.** Detailed Methods
eReferences
**eTable 1.** Normalized Root Mean Squared Errors (RMSEs) for Each Age-Specific Model, Expected Versus Observed Values Prior to March 1, 2020 **eTable 2.** Rate Ratios (95% CIs) of Observed Versus Expected RSV-Related Hospitalizations and Intensive Care Unit (ICU) Admissions Among Ontario Children <5 Years of Age During the 2021/22 and 2022/23 Seasons

Supplement 2.
Data Sharing Statement
