# Supplementary material for: Pediatric Respiratory Syncytial Virus Hospitalizations, 2017-2023
Source: JAMA Netw Open. 2024 Jun 11;7(6):e2416077. doi: 10.1001/jamanetworkopen.2024.16077 (PMC11167505; doi:10.1001/jamanetworkopen.2024.16077)
Supplement: Supplement 1. — eMethods. Detailed Methods eReferences eTable 1. Normalized Root Mean Squared Errors (RMSEs) for Each Age-Specific Model, Expected Versus Observed Values Prior to March 1, 2020 eTable 2. Rate Ratios (95% CIs) of Observed Versus Expected RSV-Related Hospitalizations and Intensive Care Unit (ICU) Admissions Among Ontario Children <5 Years of Age During the 2021/22 and 2022/23 Seasons [file jamanetwopen-e2416077-s001.pdf]

Supplementary Online Content

Fitzpatrick T, Buchan SA, Mahant S, et al. Pediatric respiratory syncytial virus hospitalizations, 2017-2023. *JAMA Netw Open*. 2024;7(6):e2416077. doi:10.1001/jamanetworkopen.2024.16077

eMethods. Detailed Methods

eReferences

eTable 1. Normalized Root Mean Squared Errors (RMSEs) for Each Age-Specific Model, Expected Versus Observed Values Prior to March 1, 2020

eTable 2. Rate Ratios (95% CIs) of Observed Versus Expected RSV-Related Hospitalizations and Intensive Care Unit (ICU) Admissions Among Ontario Children <5 Years of Age During the 2021/22 and 2022/23 Seasons

This supplementary material has been provided by the authors to give readers additional information about their work.

© 2024 Fitzpatrick T et al. *JAMA Network Open.*

eMethods. Detailed Methods

Datasets included were linked using unique encoded identifiers and analyzed at ICES.(1) All children under five years of age living in Ontario during the study period were identified from the Registered Persons Databases (RPDB), a population registry of individuals eligible for the provincial health insurance program: the Ontario Health Insurance Plan (OHIP). All permanent Ontarian residents are eligible for OHIP.

RSV-related hospitalizations were identified using a highly sensitive and specific health administrative data, previously validated in the Ontario child population and found to have both high sensitivity and specificity compared to laboratory-confirmed RSV admissions.(2) Specifically, any admission with the following International Classification of Diseases, 10th revision (ICD-10) diagnostic codes identified anywhere on the discharge abstract was considered to be RSV-related: J12.1 (RSV pneumonia), J20.5 (Acute bronchitis due to RSV), J21.0 (Acute bronchiolitis due to RSV), and B97.4 (RSV as the cause of disease classified elsewhere).

Socio-demographic and health status characteristics of children were determined at index (i.e., RSV admission date or July 1 of a given year) using linked health and census-based databases. Specifically, age at admission and postal code were determined from the Canadian Institute for Health Information (CIHI) Discharge Abstract Database (DAD). Rurality was determined from postal code and defined as communities with fewer than 10,000 residents. Neighbourhood-level marginalization was determined at the level of dissemination area, representing approximately 400-700 residents each, using the census-based 2016 Ontario Marginalization (ON-Marg) Material Resources Index.(3) In brief, the Material Resources Index considers the following census-based neighbourhood characteristics associated with material deprivation: percent of families that are lone-parent; percent of population that is aged 25+ years without a certificate, degree, or diploma; proportion of family income that is obtained from government transfer payments; percent of individuals aged 15+ years that are unemployed; percent of people living below the Low Income Measure (i.e., below the low income cut-off); and percent of residential households in fair or poor condition (i.e., in need of repair).For children born in Ontario, prematurity was determined from their birth record using the MOMBABY birth registry. Diagnosis of a complex medical condition (CMC) was defined using an approach described in previous work by CIHI,(4) which considers a child as having a CMC if one or more discharge diagnoses related to any CMC is identified from birth until index date in the relevant provincial health service encounter databases: DAD, CIHI's Same Day Surgery (SDS) database, and select disease registries (e.g., organ transplants). Palivizumab eligibility was determined based on Ontario's 2022-23 RSV Prophylaxis for High Risk Infants Program eligibility criteria, using an administrative data algorithm described previously.(5,6) As certain characteristics used to determine eligibility for the provincial prophylaxis program are beyond the scope of health administrative data (e.g., daycare attendance), children were classified as clearly eligible (i.e., met clearly identifiable clinical characteristics such as extreme prematurity or diagnosis of

© 2024 Fitzpatrick T et al. *JAMA Network Open.*

specific congenital heart or lung conditions), possibly eligible (i.e., more information was needed), and ineligible for the provincial palivizumab program (i.e., clearly did not meet any criteria); clearly and possibly eligible children were combined when calculating rate ratios due to small cell sizes. Data capturing palivizumab receipt are not captured in provincial electronic health administrative databases and, thus, were not available for analysis.

© 2024 Fitzpatrick T et al. *JAMA Network Open.*

eReferences

1. ICES. ICES Data Dictionary [Internet]. 2022 [cited 2022 Aug 8]. Available from: https://datadictionary.ices.on.ca/Applications/DataDictionary/Default.aspx

2. Pisesky A, Benchimol EI, Wong CA, Hui C, Crowe M, Belair MA, et al. Incidence of Hospitalization for Respiratory Syncytial Virus Infection amongst Children in Ontario, Canada: A Population-Based Study Using Validated Health Administrative Data. PLoS One. 2016;11(3):e0150416.

3. PHO. Public Health Ontario. 2016 [cited 2023 Aug 10]. Ontario Marginalization Index (ON-Marg). Available from: https://www.publichealthontario.ca/en/Data-and-Analysis/Health-Equity/Ontario-Marginalization-Index

4. CIHI. Children and youth with medical complexity in Canada [Internet]. 2020 [cited 2023 Sep 7]. Available from: https://www.cihi.ca/en/children-and-youth-with-medical-complexity-in-canada

5. Ontario Ministry of Health. Respiratory Syncytial Virus Prophylaxis for High-Risk Infants Program-Publicly Funded Drug Programs - Health Care Professionals - MOH [Internet]. Government of Ontario, Ministry of Health and Long-Term Care; 2022 [cited 2022 Aug 8]. Available from: https://www.health.gov.on.ca/en/pro/programs/drugs/funded_drug/fund_respiratory.aspx

6. Fitzpatrick T, McNally JD, Stukel TA, Kwong JC, Wilton AS, Fisman D, et al. Palivizumab's real-world effectiveness: a population-based study in Ontario, Canada, 1993-2017. Arch Dis Child. 2021 Feb;106(2):173–9.

© 2024 Fitzpatrick T et al. *JAMA Network Open.*

eTable 1. Normalized root mean squared errors (RMSEs) for each age-specific model, expected versus observed values prior to March 1, 2020

**Age-specific hospitalization rates**		**Age-specific ICU admission rates**	
**Age group**	**Normalized RMSE**	**Age group**	**Normalized RMSE**
0-1 month	0.091147	0-1 month	0.145721
2-3 months	0.086919	2-5 months	0.128125
4-5 months	0.094521	6-11 months	0.136735
6-11 months	0.098262	12-23 months	0.131568
12-23 months	0.079912	24-59 months	0.136135
24-59 months	0.071893		

© 2024 Fitzpatrick T et al. *JAMA Network Open.*

eTable 2. Rate ratios (95% CIs) of observed versus expected RSV-related hospitalizations and intensive care unit (ICU) admissions among Ontario children <5 years of age during the 2021/22 and 2022/23 seasons

Characteristics	Rate Ratio (95% CI)			
	RSV admissions		ICU admissions	
	2021/22	2022/23	2021/22	2022/23
Age at admission				
<1 months	0.79 (0.51, 1.24)	1.40 (0.86, 2.26)	0.78 (0.27, 2.25)	1.80 (0.54, 5.96)
2-3 months[a]	0.53 (0.28, 1.02)	1.16 (0.60, 2.25)	0.52 (0.25, 1.06)	1.75 (0.79, 3.90)
4-5 months[a]	0.66 (0.28, 1.54)	1.67 (0.91, 3.08)		
6-11 months	0.52 (0.32, 0.86)	1.58 (0.98, 2.57)	0.77 (0.24, 2.48)	2.53 (0.74, 8.68)
12-23 months	0.42 (0.28, 0.64)	1.12 (0.73, 1.73)	0.29 (0.12, 0.70)	1.30 (0.58, 2.89)
24-59 months	0.66 (0.46, 0.94)	1.90 (1.35, 2.66)	0.96 (0.43, 2.12)	3.83 (1.91, 7.66)
Sex				
Female	0.60 (0.46, 0.79)	1.45 (1.13, 1.87)	0.57 (0.26, 1.28)	1.38 (0.76, 2.50)
Male	0.63 (0.39, 1.02)	1.52 (0.84, 2.75)	0.76 (0.37, 1.58)	2.63 (1.28, 5.40)
Rural residence				
Urban	0.60 (0.44, 0.81)	1.46 (1.03, 2.07)	0.71 (0.39, 1.31)	2.16 (1.29, 3.64)
Rural	0.72 (0.34, 1.55)	1.66 (0.75, 3.67)	0.42 (0.09, 1.86)	0.91 (0.14, 6.00)
Neighbourhood marginalization quintile				
1 (least marginalized)[a]	0.53 (0.23, 1.24)	1.18 (0.52, 2.67)	0.71 (0.39, 1.31)	2.16 (1.29, 3.64)
2[a]	0.58 (0.36, 0.92)	1.19 (0.70, 2.03)		
3	0.61 (0.31, 1.17)	1.58 (0.95, 2.62)	0.42 (0.09, 1.86)	0.91 (0.14, 6.00)
4	0.74 (0.31, 1.76)	2.02 (0.96, 4.25)	0.71 (0.39, 1.31)	2.16 (1.29, 3.64)
5 (Most marginalized)	0.63 (0.33, 1.20)	1.54 (0.65, 3.63)	0.42 (0.09, 1.86)	0.91 (0.14, 6.00)
Born preterm, <37 weeks gestation	0.58 (0.29, 1.16)	1.31 (0.56, 3.05)	0.84 (0.24, 2.93)	1.57 (0.36, 6.80)
Any complex medical condition (CMC)	0.58 (0.38, 0.89)	1.28 (0.84, 1.97)	0.68 (0.28, 1.65)	1.39 (0.62, 3.11)
Palivizumab eligible	1.02 (0.32, 3.32)	2.24 (0.62, 8.16)	0.70 (0.12, 4.17)	1.15 (0.17, 7.51)

a Categories collapsed for ICU admissions due to small cell sizes.

© 2024 Fitzpatrick T et al. *JAMA Network Open.*
