# Supplementary material for: Pediatric Respiratory Syncytial Virus Hospitalizations, 2017-2023
Source: JAMA Netw Open. 2024 Jun 11;7(6):e2416077. doi: 10.1001/jamanetworkopen.2024.16077 (PMC11167505; doi:10.1001/jamanetworkopen.2024.16077)
Supplement: Supplement 2. — Data Sharing Statement [file jamanetwopen-e2416077-s002.pdf]

Data Sharing Statement

Fitzpatrick. Respiratory Syncytial Virus Hospitalizations in 2017-2023 in Ontario, Canada. *JAMA Netw Open*. Published June 11, 2024. doi:10.1001/jamanetworkopen.2024.16077

Data
Data available: Yes
Data types: Deidentified participant data
How to access data: The data set from this study is held securely in coded form at ICES. While legal data sharing agreements between ICES and data providers (eg, health care organizations and government) prohibit ICES from making the data set publicly available, access may be granted to those who meet pre-specified criteria for confidential access, available at www.ices.on.ca/DAS (email: das@ices.on.ca). The full data set creation plan and underlying analytic code are available from the authors upon request, understanding that the computer programs may rely upon coding templates or macros that are unique to ICES and are therefore either inaccessible or may require modification.
When available: With publication

Supporting Documents
Document types: None

Additional Information
Who can access the data: The data set from this study is held securely in coded form at ICES. While legal data sharing agreements between ICES and data providers (eg, health care organizations and government) prohibit ICES from making the data set publicly available, access may be granted to those who meet pre-specified criteria for confidential access, available at www.ices.on.ca/DAS (email: das@ices.on.ca). The full data set creation plan and underlying analytic code are available from the authors upon request, understanding that the computer programs may rely upon coding templates or macros that are unique to ICES and are therefore either inaccessible or may require modification.
Types of analyses: The data set from this study is held securely in coded form at ICES. While legal data sharing agreements between ICES and data providers (eg, health care organizations and government) prohibit ICES from making the data set publicly available, access may be granted to those who meet pre-specified criteria for confidential access, available at www.ices.on.ca/DAS (email: das@ices.on.ca). The full data set creation plan and underlying analytic code are available from the authors upon request, understanding that the computer programs may rely upon coding templates or macros that are unique to ICES and are therefore either inaccessible or may require modification.
Mechanisms of data availability: The data set from this study is held securely in coded form at ICES. While legal data sharing agreements between ICES and data providers (eg, health care organizations and government) prohibit ICES from making the data set publicly available, access may be granted to those who meet pre-specified criteria for confidential access, available at www.ices.on.ca/DAS (email: das@ices.on.ca). The full data set creation plan and underlying analytic code are available from the authors upon request, understanding that the computer programs may rely upon coding templates or macros that are unique to ICES and are therefore either inaccessible or may require modification.
